# Prevalence of Depression Among Urban and Rural Residents of Saudi Arabia Compared With Other Gulf Cooperation Council Countries: A Systematic Review

**DOI:** 10.7759/cureus.90321

**Published:** 2025-08-17

**Authors:** Tameem A Alhomaid, Omar Alburaidi, Nahla Garallah, Sultanah Alghofaili, Roqayya M Alhayyani, Bashayr Alkhalifah, Anwar Albalawi, Sajidah S Alshanqyti, Ranem Allaboon

**Affiliations:** 1 Family Medicine, Qassim Health Cluster, Buraydah, SAU; 2 Unaizah College of Medicine and Medical Sciences, Qassim University, Buraydah, SAU; 3 Family Medicine, King Saud Medical City, Riyadh, SAU; 4 College of Medicine, King Khalid University, Abha, SAU; 5 College of Medicine, Princess Nourah Bint Abdulrahman University, Riyadh, SAU; 6 Family Medicine, Tabuk Health Cluster, Tabuk, SAU; 7 Family and Community Medicine, Ministry of Health Holdings, Riyadh, SAU; 8 Family Medicine, King Fahad Medical City, Riyadh, SAU

**Keywords:** depression, gcc countries, mental health prevalence, saudi arabia, systematic review, urban-rural disparities

## Abstract

Depression is a leading cause of disability worldwide, with significant variations in prevalence across urban and rural populations. In the Gulf Cooperation Council (GCC) countries, rapid urbanization and socioeconomic changes have introduced new mental health challenges. However, comprehensive data on depression disparities between urban and rural residents remain limited. This systematic review aims to explore depression prevalence in Saudi Arabia and other GCC nations, examining associated factors and regional variations.

Following Preferred Reporting Items for Systematic Reviews and Meta-Analyses (PRISMA) guidelines, we conducted a systematic search of PubMed, MEDLINE, PsycINFO, Scopus, and Web of Science for studies published between 2010 and 2024. Studies published between 2010 and 2024 were included if they assessed depression prevalence among adults in urban or rural settings within GCC countries using validated diagnostic tools. Studies were excluded if they focused on narrow subpopulations or lacked clear geographic classification. Data were extracted independently by two reviewers, and study quality was assessed using the Newcastle-Ottawa Scale for observational studies and Assessment of Multiple Systematic Reviews 2 for reviews.

Twenty-four studies were included, with 18 from Saudi Arabia, four from the UAE, two from Oman, and one from Qatar. No studies from Bahrain or Kuwait met the inclusion criteria. Prevalence ranged widely: 2.1-77.8% in Saudi Arabia, 2.1-21.1% in the UAE, and 8.1-21.7% in Oman. Rural-specific data were scarce, though indirect evidence suggested higher rates in rural Saudi Arabia (e.g., 62.3% in northern regions). Women, younger adults in Qatar, older adults in Saudi Arabia, and individuals with lower socioeconomic status consistently showed higher depression rates. Stigma and underdiagnosis (74% undetected cases in Saudi Arabia) were key barriers.

Depression prevalence in the GCC varies significantly by country, urbanization level, and demographic factors. The lack of rural-specific data and studies from Bahrain and Kuwait highlights critical research gaps. Culturally tailored interventions, improved mental health infrastructure, and anti-stigma campaigns are urgently needed, particularly for women and rural populations. Future research should standardize measurement tools and prioritize disaggregated urban-rural analyses to guide equitable policy-making.

## Introduction and background

Depression is a leading cause of disability worldwide, with significant impacts on individuals’ quality of life, productivity, and overall well-being [1,2]. Globally, depressive disorders rank among the top contributors to the burden of disease, and their prevalence is rising in many regions, including the Gulf Cooperation Council (GCC) countries [3,4]. In Saudi Arabia, depression ranked fifth among the top causes of death and disability in 2019. It affects more than a third of the adult population, according to recent systematic reviews and meta-analyses [4]. This high prevalence has far-reaching implications not only for individual health but also for broader socioeconomic development, especially as the majority of the Saudi population falls within the working-age group of 18-60 years [4].

The rapid pace of urbanization and socioeconomic transformation in Saudi Arabia and other GCC countries has introduced new challenges and stressors that may contribute to the mental health burden. Urban environments, characterized by increased population density, lifestyle changes, and evolving social structures, have been associated with higher rates of mental disorders, including depression [5,6]. Findings from the Saudi National Mental Health Survey (SNMHS) indicate that mental disorders, particularly mood and anxiety disorders, are more prevalent in urban centers such as Riyadh compared to rural areas. Nearly one-third of SNMHS respondents reported a mental disorder, with almost half of these cases classified as severe, underscoring the urgent need for comprehensive mental health strategies [7].

Despite the growing recognition of depression as a significant health issue, there is considerable heterogeneity in reported prevalence rates across different regions, populations, and methodologies within Saudi Arabia. Studies have documented a wide range of depression prevalence, from as low as 8.6% in some regions to as high as 88.9% in others, reflecting differences in study design, assessment tools, and sociodemographic factors [4]. Systematic reviews estimate a pooled prevalence of depression among Saudi adults at approximately 37%, with higher rates observed among women, single individuals, those with lower education levels, and residents of the northern and southern regions of the country [4]. Risk factors such as financial difficulties, poor housing conditions, chronic health problems, and lack of social support further compound the vulnerability to depression [4,8].

When compared to other GCC countries, the prevalence of depression in Saudi Arabia appears notably higher. For instance, studies have reported prevalence rates of 4.2% to 6.6% in Qatar, 4.0% to 7.4% in Iraq, 12.5% to 28.6% in the United Arab Emirates (UAE), and 27.7% among university students in Oman [9-12]. These disparities may be attributed to variations in socioeconomic conditions, cultural attitudes toward mental health, and the availability of mental health services across the region.

Given the huge burden of depression and the observed differences between urban and rural populations, there is a critical need for systematic evidence to inform effective prevention, early detection, and management strategies tailored to the unique contexts of Saudi Arabia and the wider GCC. This systematic review aims to synthesize current knowledge on the prevalence of depression among urban and rural residents in Saudi Arabia and to compare these findings with data from other GCC countries. By identifying patterns, risk factors, and gaps in the literature, this review seeks to support the development of targeted mental health policies and interventions that address the diverse needs of populations across the region.

## Review

Study design

This systematic review was conducted following the Preferred Reporting Items for Systematic Reviews and Meta-Analyses (PRISMA) guidelines [13]. This review synthesized existing evidence on the prevalence of depression among urban and rural residents in Saudi Arabia and other GCC countries, including the UAE, Oman, Qatar, Bahrain, and Kuwait.

Literature search

The guiding research question for this review was, "What is the prevalence of depression among urban and rural residents in Saudi Arabia and other GCC countries, and what factors are associated with these prevalence rates?" To address this question, a comprehensive search strategy was used to identify relevant studies from electronic databases, including PubMed, MEDLINE, PsycINFO, Google Scholar, Scopus, and Web of Science.

The search incorporated both keywords and Medical Subject Headings terms related to population descriptors (e.g., "Urban," "Rural," "Residents"), mental health conditions (e.g., "Depression," "Depressive Disorder," "Mental Health"), geographic focus (e.g., "Saudi Arabia," "UAE," "GCC"), and study type (e.g., "Prevalence," "Epidemiology," "Cross-Sectional," "Survey"). Boolean operators such as AND, OR, and NOT were used to refine the search queries. This search string is an example of the strings used: ("Depression" OR "Depressive Disorder") AND ("Urban" OR "Rural") AND ("Saudi Arabia" OR "GCC" OR "UAE" OR "Oman" OR "Qatar" OR "Bahrain" OR "Kuwait") AND ("Prevalence" OR "Epidemiology" OR "Cross-Sectional Study").

Study selection and eligibility criteria

Studies were selected based on the population, exposure, comparison, and outcomes (PECO) framework [14]. Eligible studies included adults aged 18 years and above residing in urban or rural settings within Saudi Arabia or other GCC countries. While there was no specific exposure under investigation, comparisons between urban and rural prevalence rates were considered when available. The primary outcome of interest was the prevalence of depression, assessed using validated screening or diagnostic tools such as the Patient Health Questionnaire-9 (PHQ-9) [15], Beck Depression Inventory (BDI) [16], or Composite International Diagnostic Interview (CIDI) [17].

To be included, studies had to be original empirical research published in English, report on depression prevalence in urban and/or rural populations, use validated diagnostic tools, and be conducted between 2010 and 2024 to ensure contemporary relevance. Studies were excluded if they focused solely on specific subpopulations unless they could be generalized to broader urban/rural populations. Additionally, case reports, editorials, non-empirical studies, and studies lacking clear geographic classification were excluded.

Data extraction

Two reviewers independently performed data extraction, and any discrepancies were resolved through discussion or consultation with a third reviewer. Key data extracted included study characteristics (author, year, country, study design, and sample size), population details (urban or rural classification and demographics such as age, gender, and socioeconomic status), outcome measures (prevalence rates, assessment tools used, and associated factors like income or marital status), and key findings, particularly urban-rural comparisons and significant predictors of depression.

Quality assessment and risk of bias

The quality of included studies and the potential risk of bias were evaluated using appropriate tools. For observational studies, the Newcastle-Ottawa Scale (NOS) [18] was used to assess domains such as selection, comparability, and outcome assessment. For systematic reviews and meta-analyses included in the synthesis, the Assessment of Multiple Systematic Reviews 2 (AMSTAR-2) tool [19] was employed to evaluate methodological quality. Specific domains evaluated across all studies included selection bias (e.g., representativeness of urban or rural samples), measurement bias (e.g., use of validated tools), and reporting bias (e.g., completeness in reporting geographic-specific prevalence rates). The NOS and AMSTAR-2 are publicly available tools developed by the University of Ottawa and McMaster University, respectively, and can be freely used without specific permission.

Statistical analysis and synthesis

**Figure 1:**
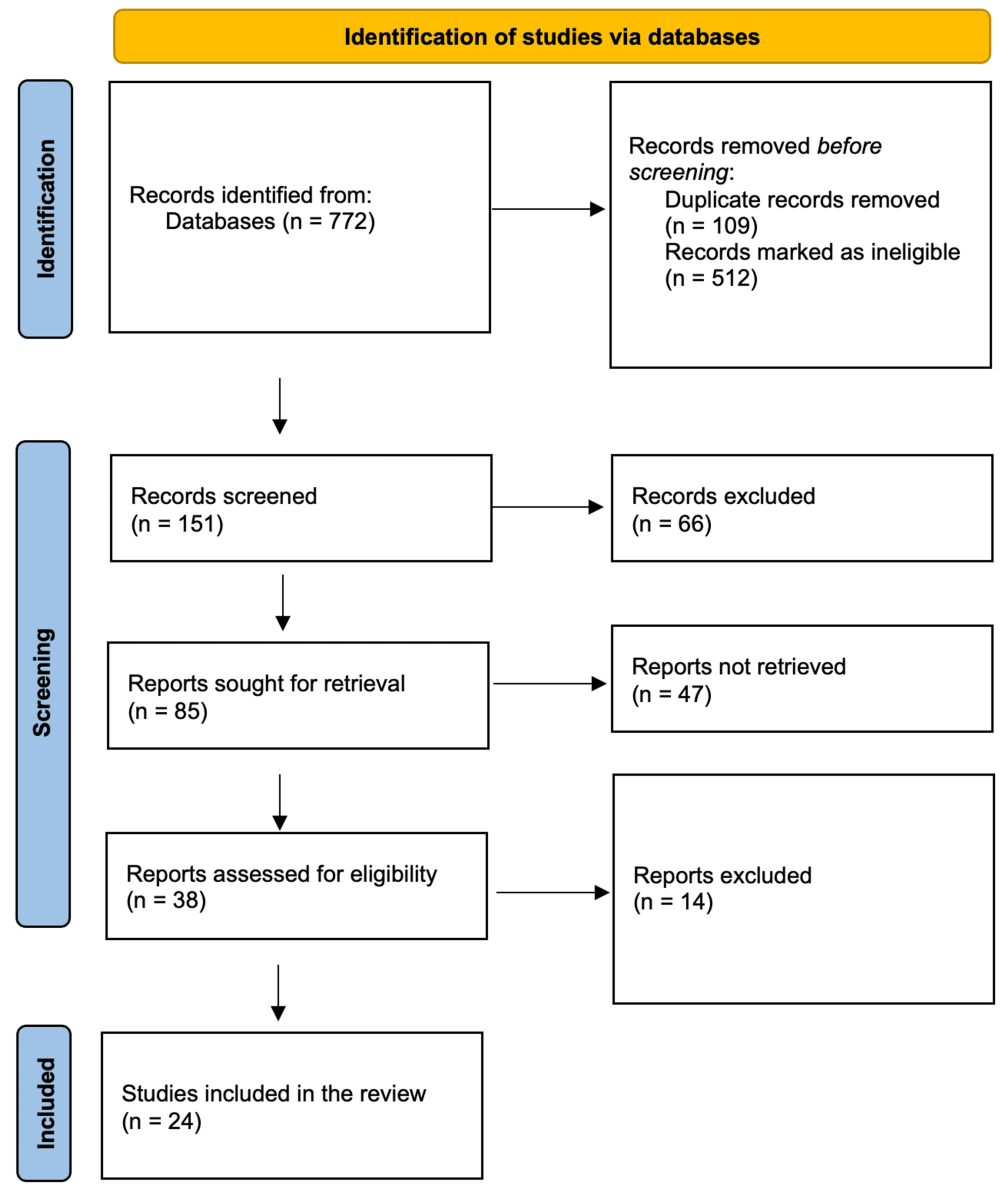
PRISMA flowchart showing the study selection process PRISMA: Preferred Reporting Items for Systematic Reviews and Meta-Analyses

Due to substantial heterogeneity in study designs, populations, and measurement tools, a meta-analysis was deemed inappropriate. Instead, a narrative synthesis approach was adopted. This included a descriptive summary of the prevalence rates across countries and settings, subgroup analyses stratified by demographic factors such as gender, age, and socioeconomic status, and a qualitative exploration of themes such as stigma, healthcare access, and cultural influences on depression prevalence. The study selection process was visually summarized using a PRISMA flow diagram (Figure 1), which detailed that out of 772 initially identified records, 151 remained after duplicate removal and initial identification of ineligible studies. From these, 38 full-text articles were screened, resulting in 24 studies being included in the final synthesis.

Results

The systematic review included 24 studies examining the prevalence of depression among urban and rural residents in Saudi Arabia and other GCC countries.

The majority of the studies (18 out of 24) were conducted in Saudi Arabia, highlighting a dominant mental health research focus on this country within the GCC region. Four studies were carried out in the UAE, two in Oman, and one in Qatar. Notably, no studies from Bahrain or Kuwait met the inclusion criteria, highlighting gaps in research from these countries.

Fourteen studies focused exclusively on urban populations, while 11 included both urban and rural residents. None of the studies examined rural populations in isolation, suggesting a potential oversight in understanding the unique mental health challenges faced by rural communities.

The sample sizes varied significantly across the studies. Fourteen studies had fewer than 1,000 participants, seven ranged between 1,000 and 5,000 participants, and three studies stood out with large sample sizes exceeding 5,000 participants. The largest study, conducted by BinDhim et al. [20], included 16,513 participants, providing robust data on mental health trends in Saudi Arabia during the COVID-19 pandemic.

The PHQ-9 was the most frequently used tool, employed in 11 studies, while the PHQ-2 and PHQ-9 (both publicly and freely available tools) were used together in two studies. The BDI/BDI-II was utilized in three studies. Three studies used their own designed, tested, and validated questionnaires. Other tools, such as the World Health Survey tool, CIDI 3.0, Seasonal Pattern Assessment Questionnaire (SPAQ), and Depression Anxiety Stress Scales-21 (DASS-21), were each used in single studies. The reliance on the PHQ-9 underscores its widespread acceptance for depression screening in the region, though the use of varied tools may introduce heterogeneity in prevalence estimates. Table 1 shows the characteristics of the included studies.

**Table 1:** Characteristics of the included studies PHQ-9: Patient Health Questionnaire-9, PHQ-2: Patient Health Questionnaire-2, BDI: Beck Depression Inventory, BDI-II: Beck Depression Inventory-II, CIDI 3.0: Composite International Diagnostic Interview version 3.0, DASS-21: Depression, Anxiety, and Stress Scale-21, SPAQ: Seasonal Pattern Assessment Questionnaire, UAE: United Arab Emirates, PHC: Primary Health Care, MHSS: Mental Health Surveillance System

Authors	Title	Design	Location/country	Population type	Sample Size	Assessment tools
Alruwaili and Alanazi [21], 2019	Prevalence and associated factors of depressive symptoms among adults with overt hypothyroidism on treatment in Riyadh: a cross-sectional study	Cross-sectional study	Riyadh, Saudi Arabia	Urban	369	PHQ-9 (Arabic version)
Abdullatif et al. [22], 2021	Prevalence of depressive disorders and associated factors among adult population of Dubai 2019	Cross-sectional study	Dubai, UAE	Urban	2,244	PHQ-9
Al Harrasi and Masad [23], 2017	Predictors of depression among adult Omani women in Wilayat of Rustaq	Cross-sectional study	Rustaq, Oman	Urban and Rural	240	BDI-II (Arabic version)
Al-Atawi et al. [24], 2016	Prevalence and determinants of depression among type 2 diabetic patients in Tabuk City, Saudi Arabia	Cross-sectional study	Tabuk, Saudi Arabia	Urban	221	PHQ-9 (Arabic version)
Al-Dabal et al. [25], 2015	Magnitude of depression problem among primary care consumers in Saudi Arabia	Cross-sectional study	Al Khobar, Saudi Arabia	Urban	850	PHQ-9 (Arabic version)
Al-Lugmani [2], 2014	Depression among hypertensive patients at al-Hejrah PHC Center Makkah Al-Mukarramah	Cross-sectional study	Makkah, Saudi Arabia	Urban	54	BDI (Arabic version)
Al-Salmani et al. [26], 2015	Characterization of depression among patients at urban primary healthcare centers in Oman	Cross-sectional study	Muscat, Oman	Urban	2,005	PHQ-9
AlHamad and Alamri [27], 2021	The association between social media use and depressive symptoms among adults in Riyadh, Saudi Arabia	Cross-sectional study	Riyadh, Saudi Arabia	Urban	467	PHQ-9
Alhabeeb et al. [28], 2023	National screening for anxiety and depression in Saudi Arabia 2022	Cross-sectional study	All regions, Saudi Arabia	Urban and Rural	6,015	PHQ-9
Alkaabi et al. [29], 2022	The prevalence and correlates of depression among patients with chronic diseases in the United Arab Emirates	Cross-sectional study	Al-Ain, UAE	Urban	417	PHQ-9
Alqahtani et al. [30], 2023	Prevalence of seasonal affective disorder among primary health care attendees in eastern Riyadh-a cross-sectional study	Cross-sectional study	Eastern Riyadh, Saudi Arabia	Urban	232	SPAQ
Alshehri et al. [31], 2023	Study to determine the epidemiology of treatment-resistant depression among the Saudi Arabian population: a cross-sectional study	Cross-sectional study	Abha City, Saudi Arabia	Urban	651	Own-designed questionnaire
Alsulaimani [32], 2020	Risk factors of depression among Saudi females	Cross-sectional study	Abha, Saudi Arabia	Urban	317	PHQ-2 and PHQ-9
Alsuwaidan et al. [33], 2024	Prevalence of depression in postmyocardial infarction patients in a tertiary care center in Riyadh	Cross-sectional study	Riyadh, Saudi Arabia	Urban	249	PHQ-2 and PHQ-9
Altwaijri et al. [7], 2023	Dual burden of chronic physical conditions and mental disorders: findings from the Saudi National Mental Health Survey	Cross-sectional study	All regions, Saudi Arabia	Urban and Rural	4,001	CIDI 3.0
Alzughbi et al. [34], 2020	Diabetes-related distress and depression in Saudis with type 2 diabetes	Cross-sectional study	Jazan, Saudi Arabia	Urban and Rural	300	PHQ-9
BinDhim et al. [20], 2021	Saudi Arabia Mental Health Surveillance System (MHSS): mental health trends amid COVID-19 and comparison with pre-COVID-19 trends	Cross-sectional study	All regions, Saudi Arabia	Urban and Rural	16,513	PHQ-9 (Arabic version)
Ghuloum et al. [35], 2011	Prevalence of mental disorders in the adult population attending primary health care settings in Qatari population	Cross-sectional study	Qatar	Urban	1,660	Own-designed questionnaire
Jareebi et al. [36], 2024	Common mental health conditions and self-stigma in Saudi adults: implications for promotion and intervention	Cross-sectional study	Jazan, Saudi Arabia	Urban and Rural	1,056	DASS-21
Madkhali et al. [37], 2019	Prevalence and associated factors of depression among patients with diabetes at Jazan Province, Saudi Arabia: a cross-sectional study	Cross-sectional study	Jazan Province, Saudi Arabia	Urban and Rural	480	BDI-II (Arabic version)
Nour et al. [4], 2023	Prevalence of depression and associated factors among adults in Saudi Arabia: systematic review and meta-analysis (2000–2022)	Systematic review and meta-analysis	All regions, Saudi Arabia	Urban and Rural	25,814	Various
Odah et al. [38], 2024	Prevalence of depression among the adult population in southwestern Saudi Arabia: a cross-sectional, community-based study	Cross-sectional study	Al-Qunfudah, Saudi Arabia	Urban and Rural	1,036	PHQ-9 (Arabic version)
Salim et al. [39], 2020	Mental health reflections and self-rating at population contexts and paradigms	Cross-sectional study	Dubai, UAE	Urban	2,532 families	World Health Survey Tool
Shahda et al. [40], 2016	Clinical patterns of mood disorders in a sample of mood disorder patients in the United Arab Emirates	Cross-sectional study	UAE	Urban and Rural	~500	Own-designed questionnaire

Table 2 shows variability in the reported prevalence of depression across studies conducted in Saudi Arabia and other GCC countries. In Saudi Arabia, depression prevalence ranged widely, from 12.7% in national community samples to over 64% in specific urban clinical populations, highlighting regional, methodological, and population-specific differences. Urban populations frequently reported higher prevalence, with notably elevated rates among those with chronic conditions or post-myocardial infarction. While the UAE and Oman showed generally lower prevalence (8.1%-39%), Qatar reported a single estimate of 13.5%. The pooled estimate from the meta-analysis of Saudi studies was approximately 30%.

**Table 2:** Summary of depression prevalence outcomes in included studies MI: myocardial infarction, COVID-19: coronavirus disease 2019

Authors	Reported depression prevalence (%)
Alruwaili and Alanazi [21], 2019	33.10%
Abdullatif et al. [22], 2021	12.50%
Al Harrasi and Masad [23], 2017	27.00%
Al-Atawi et al. [24], 2016	49.30%
Al-Dabal et al. [25], 2015	41.70%
Al-Lugmani [2], 2014	64.80%
Al-Salmani et al. [26], 2015	8.10%
AlHamad and Alamri [27], 2021	47.30%
Alhabeeb et al. [28], 2023	12.70%
Alkaabi et al. [29], 2022	39.00%
Alqahtani et al. [30], 2023	18.5% (seasonal affective disorder)
Alshehri et al. [31], 2023	22.7% (treatment-resistant depression)
Alsulaimani [32], 2020	25.90%
Alsuwaidan et al. [33], 2024	37.3% (post-MI patients)
Altwaijri et al. [7], 2023	13.80%
Alzughbi et al. [34], 2020	34.30%
BinDhim et al. [20], 2021	23.6% (during COVID-19)
Ghuloum et al. [35], 2011	13.50%
Jareebi et al. [36], 2024	19.7% (moderate-severe depression)
Madkhali et al. [37], 2019	20.20%
Nour et al. [4], 2023	29.9% (pooled estimate)
Odah et al. [38], 2024	27.70%
Salim et al. [39], 2020	19.50%
Shahda et al. [40], 2016	20.00%

Rural prevalence data were not consistently reported in any of the studies. Although several included both urban and rural populations, none disaggregated findings by locality. However, some insights can be drawn from studies that included both urban and rural populations. For instance, Alhabeeb et al. [28] reported a national depression prevalence of 12.7% in Saudi Arabia but did not differentiate between urban and rural settings. Similarly, Madkhali et al. [37] conducted a study in Jazan Province, where 60.6% of participants were from rural areas, but did not provide comparative prevalence data between urban and rural groups.

On the other hand, Nour et al. [4] found that the northern region of Saudi Arabia, characterized by more rural populations, had a significantly higher depression prevalence (62.3%) compared to other regions. This suggests potential disparities in mental health burden between rural and urban areas, though more targeted analysis is needed. In the UAE, Shahda et al. [40] observed qualitative differences in depression symptom profiles rather than prevalence. Rural residents were more likely to report depressed mood, appetite loss, and fatigue, while urban residents more often experienced anhedonia and insomnia.

Several factors were commonly associated with higher depression rates (Table 3). Gender was frequently reported, with studies in Saudi Arabia, Oman, and Qatar all reporting higher prevalence among women. Age was also significant, though patterns differed, as older individuals had higher rates in Oman, while younger individuals were more affected in Qatar. In Saudi Arabia, lower socioeconomic status was linked to higher depression prevalence. In the UAE, being a national was associated with higher rates compared to non-nationals, suggesting the influence of social or cultural pressures. Most studies report higher depression rates among unmarried, divorced, or widowed individuals, except Al-Salmani et al. [26], who found a positive association between being married and depression (odds ratio = 1.91, P = 0.02). No studies providing depression prevalence estimates were found for Bahrain and Kuwait.

**Table 3:** Associated factors of depression in GCC populations GCC: Gulf Cooperation Council, OR: odds ratio, SAR: Saudi Riyal

Factor category	Key findings	Examples from studies	Potential explanations
Gender	Women consistently show higher depression rates	Alhabeeb et al. [28]: women had 1.472x higher odds (p < 0.001)	Cultural roles, biological differences, and limited mental health access
Age	Mixed results: higher in younger (e.g., Qatar), higher in elderly (e.g., Saudi Arabia)	Alhabeeb et al. [28]: highest prevalence (14.8%) in the 60+ age group	Younger: academic/job stress, elderly: chronic illness, loneliness
Marital status	Most studies: higher in unmarried/divorced/widowed, exception: married (Al-Salmani et al. [26])	Al-Salmani et al. [26]: married OR = 1.91 (p = 0.02)	Unmarried: social isolation, married: familial/job stress
Education level	Lower education linked to higher depression	Alhabeeb et al. [28]: no bachelor's degree OR = 0.771 (p < 0.001)	Limited job opportunities, socioeconomic disadvantage
Income status	Lower income associated with higher depression	Alhabeeb et al. [28]: income < 5,000 SAR/month OR = 0.808 (p = 0.018)	Financial stress, reduced access to healthcare
Employment status	Unemployment leads to higher depression, housewives and government employees at high risk	Multiple studies	Unemployment: financial instability, housewives: isolation, government jobs: stress
Healthcare access	Inferred as a factor in prevalence and detection	BinDhim et al. [20]: 74% undiagnosed depression	Chronic conditions: higher depression rates for individuals with chronic conditions. Low diagnosis rates among the affected

As shown by Table 4, four studies [7,20,22,28] achieved the highest NOS scores of 9, indicating a low risk of bias. These studies were characterized by large national samples, inclusion of both rural and urban populations, and robust study designs, such as pre-/post-pandemic evaluations and national surveys. Their strong performance in all three NOS domains (selection, comparability, and outcome) further reinforced their credibility.

**Table 4:** Quality and risk of bias assessment of included studies NOS: Newcastle-Ottawa Scale, AMSTAR-2: Assessment of Multiple Systematic Reviews 2

Authors	Assessment tool	Selection (max 4)	Comparability (max 2)	Outcome (max 3)	Total NOS score (max 9)	Overall risk of bias	Notes
Alruwaili and Alanazi [21], 2019	NOS	3	1	2	6	Moderate	Small sample size, urban-only focus
Abdullatif et al. [22], 2021	NOS	4	2	3	9	Low	Large sample, robust methodology
Al Harrasi and Masad [23], 2017	NOS	3	1	2	6	Moderate	Focus on women only, rural-urban mix
Al-Atawi et al. [24], 2016	NOS	2	1	2	5	High	Small sample, clinical population bias
Al-Dabal et al. [25], 2015	NOS	3	2	2	7	Moderate	Urban primary care setting
Al-Lugmani [2], 2014	NOS	2	1	1	4	High	Very small sample, single-center
Al-Salmani et al. [26], 2015	NOS	3	2	2	7	Moderate	Urban focus, validated tool
AlHamad and Alamri [27], 2021	NOS	3	1	2	6	Moderate	Social media bias, urban-only
Alhabeeb et al. [28], 2023	NOS	4	2	3	9	Low	National sample, rural-urban included
Alkaabi et al. [29], 2022	NOS	3	1	2	6	Moderate	Chronic disease bias, urban-only
Alqahtani et al. [30], 2023	NOS	3	1	2	6	Moderate	Seasonal focus, small sample
Alshehri et al. [31], 2023	NOS	2	1	2	5	High	Non-validated tool, treatment-resistant focus
Alsulaimani [32], 2020	NOS	3	1	2	6	Moderate	Female-only sample, urban
Alsuwaidan et al. [33], 2024	NOS	3	1	2	6	Moderate	Post-MI patients, single-center
Altwaijri et al. [7], 2023	NOS	4	2	3	9	Low	National survey, robust design
Alzughbi et al. [34], 2020	NOS	3	1	2	6	Moderate	Diabetes focus, rural-urban mix
BinDhim et al. [20], 2021	NOS	4	2	3	9	Low	Large national sample, pre-/post-COVID-19
Ghuloum et al. [35], 2011	NOS	3	1	2	6	Moderate	Non-validated tool, urban primary care
Jareebi et al. [36], 2024	NOS	3	2	2	7	Moderate	Rural-urban mix, stigma focus
Madkhali et al. [37], 2019	NOS	3	1	2	6	Moderate	Diabetes focus, rural-urban mix
Nour et al. [4], 2023	AMSTAR-2	-	-	-	-	Moderate	Comprehensive meta-analysis
Odah et al. [38], 2024	NOS	3	2	2	7	Moderate	Community-based, rural-urban mix
Salim et al. [39], 2020	NOS	3	1	2	6	Moderate	Urban, self-report bias
Shahda et al. [40], 2016	NOS	2	1	1	4	High	Small sample, non-validated tool

The majority of the studies (16 out of 24) were rated as having moderate risk of bias, with NOS scores ranging from 6 to 7. While these studies generally had acceptable selection criteria and outcome assessment, they frequently scored only 1 point in comparability, suggesting limited adjustment for confounding variables. Common methodological limitations included reliance on self-reported data, small or urban-only samples, and population-specific biases such as female-only or chronic disease-focused samples.

Three studies [2,24,40] were rated as having a high risk of bias, with NOS scores between 4 and 5. These studies suffered from significant limitations, including very small or clinical samples, single-center designs, and the use of non-validated tools. For instance, Al-Lugmani [2] had a total score of 4, the lowest in the group, attributed to a very small, urban-only sample from a single site. This variability underscores the need for more nationally representative, well-controlled studies using validated instruments in future research.

Discussion

This systematic review highlights significant variations in depression prevalence across urban and rural populations in GCC countries, with notable gaps in research and unique regional patterns. The findings show that Saudi Arabia has the widest reported prevalence range, likely due to differences in study populations, assessment tools, and regional disparities. The highest rate of 77.8% was reported among patients with diabetes in Tabuk City, Saudi Arabia [24]. The UAE and Oman show lower ranges, while Qatar reports a single estimate of 13.5%. No studies from Bahrain or Kuwait met the inclusion criteria, underscoring a critical research gap in these nations. The lack of disaggregated rural data is another major limitation, though indirect evidence suggests rural areas may experience higher depression rates, as seen in northern Saudi Arabia (62.3%).

These findings align with global trends in low- and middle-income countries (LMICs), where rural populations often face greater mental health burdens due to limited healthcare access and socioeconomic challenges [41]. However, they contrast with high-income countries, where urban areas typically report higher depression rates due to stressors like social isolation [42]. Though there is a lack of studies with direct urban-rural comparisons within Saudi Arabia, research demonstrated a 29% higher depression prevalence in urban compared to rural areas, particularly among lower-income groups [43]. This pattern was confirmed by a systematic review that indirectly reported a higher prevalence in Saudi rural areas [4] and may suggest similar dynamics could exist in rapidly urbanizing Saudi regions. However, local studies are needed to confirm. While direct prevalence comparisons between Saudi Arabia and neighboring GCC states are limited, with Saudi Arabia having the highest research output, previous research output analysis reveals that Saudi Arabia produced the most depression-related studies but had the lowest per capita research index (0.32/million population) [44]. Thus, Bahrain (1.45/million), Qatar (1.34/million), and Kuwait (1.28/million) showed higher research productivity relative to population size [44].

The findings may indicate a high and context-dependent burden of depression in the region, emphasizing the need for standardized diagnostic approaches and more disaggregated data, particularly for rural populations. Additionally, the diversity in assessment tools (PHQ-9, BDI, CIDI, DASS-21, and others) and target populations (general adult population vs. clinical subgroups) could further contribute to the observed differences in prevalence.

Gender disparities in depression prevalence are consistent across GCC countries, with women showing significantly higher rates. This mirrors global patterns [1,45] but may be amplified by regional factors such as restrictive gender roles and limited mental health services for women [46]. Though GCC countries have made improvements in addressing mental health challenges, women continue to experience limited access to adequate mental health services due to stigma, resource constraints, and systemic barriers [47]. Socioeconomic status also plays a critical role, with lower income and education levels consistently linked to higher depression rates. This aligns with global evidence but may be exacerbated in the GCC by labor policies like the kafala system, which disproportionately affects low-income expatriates [48,49]. Marital status findings are mixed, with most studies associating unmarried, divorced, or widowed individuals with higher depression rates. However, Al-Salmani et al. [26] found marriage itself could be a stressor (OR = 1.91), suggesting cultural and familial pressures may uniquely influence mental health in the region. Similarly, previous research showed that key risk factors of depression identified in Saudi populations were lower educational attainment, student/non-employed status, younger age (21-30 years), female gender, and single marital status [38].

A key concern is the underdiagnosis of depression, with BinDhim et al. [20] reporting that 74% of cases go undetected. This reflects pervasive stigma, where mental illness is often attributed to spiritual causes rather than medical conditions [50-52]. This supports evidence that stigma toward mental health disorders is more prevalent in Arab countries [52,53] compared to Western countries, where such barriers to help-seeking are less pronounced, with only 35% of depression cases remaining untreated [54,55]. The rapid modernization of GCC cities may also contribute to urban mental health challenges, as seen in the higher reporting of symptoms like anhedonia and insomnia [40]. In contrast, rural isolation and limited healthcare infrastructure may explain the inferred higher depression burden in these areas, a pattern observed in other LMICs like India [56], where socioeconomic and gender disadvantage factors were associated with mental disorders among women.

Climate represents an underexplored factor in GCC mental health research. While seasonal affective disorder (SAD) is well-documented in high-latitude countries, Alqahtani et al. [30] suggest extreme heat and disrupted circadian rhythms may similarly impact mood disorders in desert climates. This warrants further investigation, particularly as climate change intensifies regional temperatures. Methodological inconsistencies, such as varied assessment tools (e.g., PHQ-9 vs. BDI) and sampling biases (clinical vs. community populations), further complicate cross-study comparisons. Similar challenges have been noted in other Middle Eastern mental health research [57] and elsewhere [15]. To address these gaps, future studies should prioritize standardized measurement tools, disaggregated rural-urban data, and culturally sensitive interventions. Mobile mental health clinics, modeled after successful programs in rural Pakistan and other countries [58,59], could improve access in underserved areas. Anti-stigma campaigns involving religious leaders, as implemented in Egypt [60], may also enhance help-seeking behaviors. Additionally, research should explore the intersection of climate and mental health, building on existing work in Australia [61].

This review presents some limitations that should be considered. A major limitation of the included studies is the predominant focus on urban populations. Most studies were conducted in major cities (e.g., Riyadh, Dubai, Muscat), with very few including rural participants, and none disaggregating data by urban vs. rural settings. This limits generalizability to rural communities, where access to mental health services and social determinants of health differ significantly. The use of a wide variety of screening and diagnostic tools (e.g., PHQ-9, BDI-II, CIDI 3.0, DASS-21, and locally developed instruments) is another limitation that introduces inconsistency in depression measurement. Differences in cutoff scores, sensitivity, and cultural validity may have led to inflated or deflated prevalence estimates across studies. While some studies used robust sampling methods and national datasets, others had small, clinic-based samples or were limited to specific subpopulations (e.g., patients with diabetes or cardiovascular disease). Since depression is often underdiagnosed due to stigma, cultural beliefs, and limited awareness [50-52], some studies may have underestimated prevalence due to non-disclosure by participants or social desirability bias, particularly in face-to-face interviews. Finally, the lack of longitudinal data presents a limitation, as all included studies were cross-sectional, limiting the determination of causal relationships or trends over time. The absence of cohort studies impairs understanding of the progression and determinants of depression in these settings.

While many of the included studies relied heavily on standardized tools, which have strong psychometric properties, these instruments may not fully capture constructs like perceived social support, resilience, religiosity/spiritual well-being, community belonging, and acculturative stress, which are increasingly recognized as influential in the manifestation and reporting of depressive symptoms, particularly in Middle Eastern and Gulf populations. Future research should consider integrating such constructs alongside traditional symptom-based measures to enhance cultural sensitivity and contextual validity. Factors such as stigma, cultural idioms of distress, gender norms, and religious interpretations of mental illness can affect both self-reporting and clinical recognition of depressive symptoms. Moreover, Western-developed tools may not fully account for local expressions of psychological distress (e.g., somatic symptom emphasis), potentially leading to under- or over-estimation of prevalence. Addressing this requires adapting instruments through rigorous cross-cultural validation processes, involving bilingual translations, cognitive interviewing, and local expert input. We recommend that future studies explicitly discuss cultural bias in both measurement and interpretation and report how these issues were mitigated to improve comparability and accuracy.

Selection bias was frequent, arising from unrepresentative samples (e.g., urban-only recruitment, hospital-based sampling, or focus on specific clinical populations such as diabetic or post-MI patients). This limits the generalizability of results to the wider urban and rural populations, particularly underrepresenting rural residents and marginalized groups. Comparability bias occurred in most moderate-risk studies, where limited or no adjustment for key confounders (e.g., socioeconomic status, gender, comorbidities) could have distorted observed associations. For example, higher depression rates in women or low-income participants might partially reflect the absence of multivariable adjustment. Outcome assessment bias was also present, with substantial heterogeneity in the measurement tools used. Differences in cutoff thresholds, linguistic translations, and a lack of validation for local cultural contexts may have led to misclassification of depressive symptoms. Moreover, standard Western-developed scales may not capture newer psychosocial constructs, such as perceived social support, resilience, religiosity, and acculturative stress, which are relevant to depression manifestation in the GCC. This limitation may contribute to cultural bias and under-detection of culturally specific symptom expressions, such as somatization or religiously framed distress.

## Conclusions

Depression prevalence in Saudi Arabia and other GCC countries is a significant public health concern, with Saudi Arabia having the highest but also the widest prevalence range. The UAE, Oman, and Qatar also have higher rates compared to other developed countries. Rural areas, particularly in northern Saudi Arabia, may experience higher depression burdens due to socioeconomic disadvantages and limited healthcare access. Urban populations face unique stressors such as rapid modernization, social isolation, and lifestyle changes, resulting in distinct depressive symptom profiles. Gender disparities are highlighted, with women exhibiting higher depression rates across all studied GCC countries. Socioeconomic factors, such as lower income, unemployment, and education, are strongly associated with depression. Marital status findings are mixed, with most studies linking unmarried or divorced individuals to a higher risk. Systemic barriers to depression management include underdiagnosis and pervasive stigma. Climate-related factors, such as extreme heat, are underexplored contributors to mood disorders. Future action should include standardized research, targeted interventions, policy priorities, and expanded surveillance. While some findings align with global trends, the region’s unique socioeconomic and environmental contexts demand tailored approaches. Addressing the identified challenges requires collaboration among policymakers, healthcare providers, and community leaders to reduce stigma, enhance service delivery, and promote mental health equity across urban and rural settings.
